# Correction: Synovial gene expression after hemarthrosis differs between FVIII-deficient mice treated with recombinant FVIII or FVIII-Fc fusion protein

**DOI:** 10.1371/journal.pone.0331108

**Published:** 2025-09-04

**Authors:** Bilgimol Chumappumkal Joseph, Thomas C. Whisenant, Esther J. Cooke, Jenny Y. Zhou, Nicca Falah, Juan A. De-Pablo Moreno, Annette von Drygalski

The sixth author’s name is written incorrectly. The correct name is: Juan A. De-Pablo Moreno.

Fig 5 was uploaded incorrectly. Please see the correct Fig 5 here.

**Fig 5:**
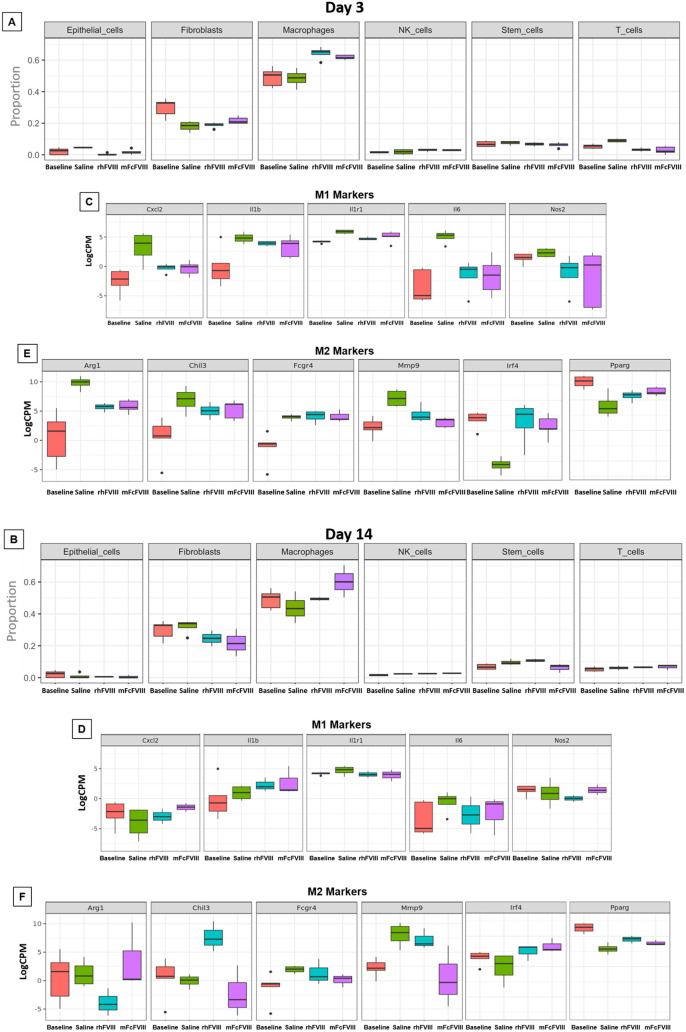
Cell type-specific gene expression in synovium on days 3 and 14. In order to identify the cell types, deconvolution analysis was used to estimate the proportions of a range of immune cells. Briefly, the IMMGEN dataset was used to generate a custom signature matrix for imputing cell fractions using the CIBERSORTx tool. Different cell populations were identified on days 3 and 14 (A and B). Differential expression levels of macrophage subtype-specific markers M1 (C and D) and M2 were determined by LogFC values (E and F). IMMGEN, Immunological genome project; LogFC, log fold change; rhFVIII, recombinant human factor VIII; mFcFVIII, mouse-specific Fc fusion factor VIII; NK cells, natural killer cells; Il6, interleukin 6; Il1b, interleukin 1b; Nos2, nitric oxide synthase 2; Cxcl2, C-X-C motif chemokine ligand 2; Ilr1, Interleukin 1 receptor type 1; Fcgr4, Fc gamma receptor 4; Irf4, Interferon regulatory factor 4; Pparg, peroxisome proliferator activated receptor gamma; Arg1, arginase-1; Chil3, chitinase-like protein 3; and Mmp9, matrix metalloproteinase 9.
