# Supplementary figures and images for: Synovial Gene expression after Hemarthrosis differs between FVIII-deficient mice treated with recombinant FVIII or FVIII-Fc Fusion Protein
Source: PLoS One. 2025 May 19;20(5):e0320322. doi: 10.1371/journal.pone.0320322 (PMC12088034; doi:10.1371/journal.pone.0320322)

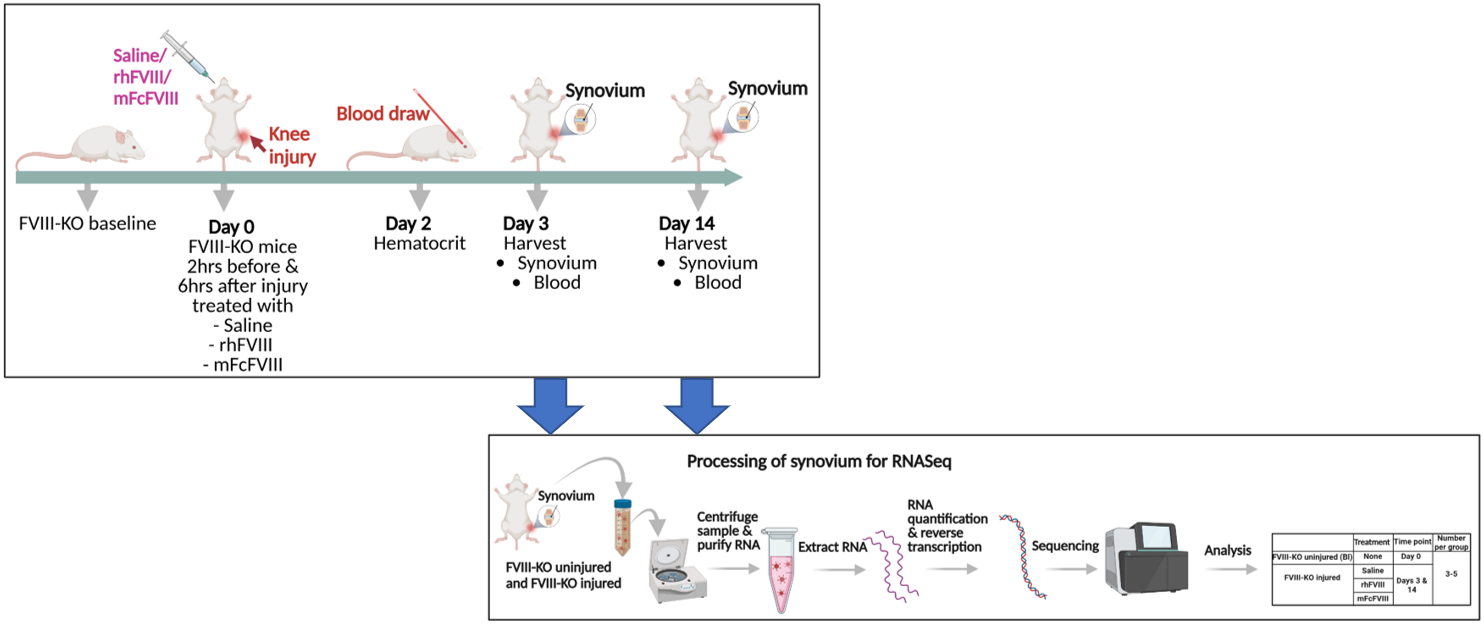

Supplement: S1 Fig — Hemarthrosis was induced in FVIII-KO mice by sub-patellar needle puncture. Saline, rhFVIII, or mFcFVIII prophylaxis was given 2 h before and 6 h after injury (n = 3 to 5 per group). The extent of intra/peri‐articular bleeding was determined by hematocrit measurement on day 2 after injury. Synovial tissue and blood were harvested at baseline, day 3, and day 14 post-injury. RNA was purified and analyzed by RNA sequencing using an Illumina NextSeq500 platform (75 bp; single‐end). The R BioConductor packages tximport, edgeR, and limma were used to estimate counts from RSEM, trimmed mean of M-values (TMM) normalization was applied, and the limma-voom method was used for differential expression analyses (criteria: adjusted p-value <0.05). FVIII-KO, Factor VIII knock-out; mFcFVIII, mouse-specific Fc-fusion FVIII; rhFVIII, recombinant human FVIII; Bl, baseline. (TIF) [file pone.0320322.s001.tif]

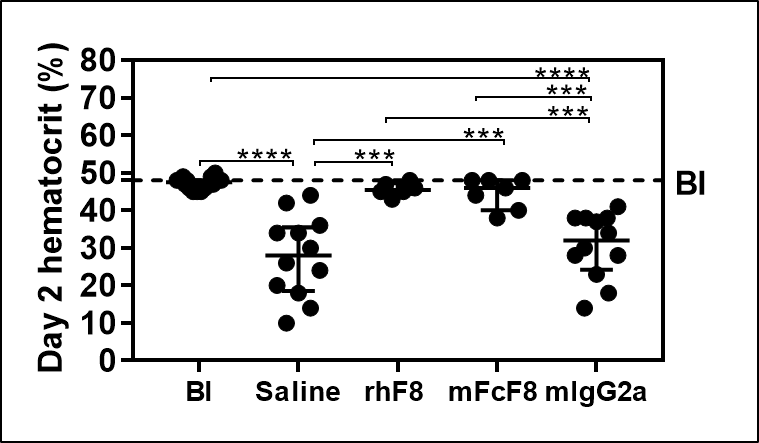

Supplement: S2 Fig — Hemarthrosis was induced by sub-patellar knee injury in FVIIIKO mice. Mice were treated with either saline (control) recombinant human factor VIII (rhFVIII/rhF8), mouse-specific Fc fusion factor VIII (mFcFVIII/rhF8), or mouse immunoglobulin (mIgG) 2a 2 hours before and 6 hours after the knee injury. Hematocrit was determined at baseline (Bl) mice and on day 2 after injury (n = 6–12 per group). Error bars represent the median with interquartile range values (***p < 0.001, ****p < 0.0001). (TIF) [file pone.0320322.s002.tif]

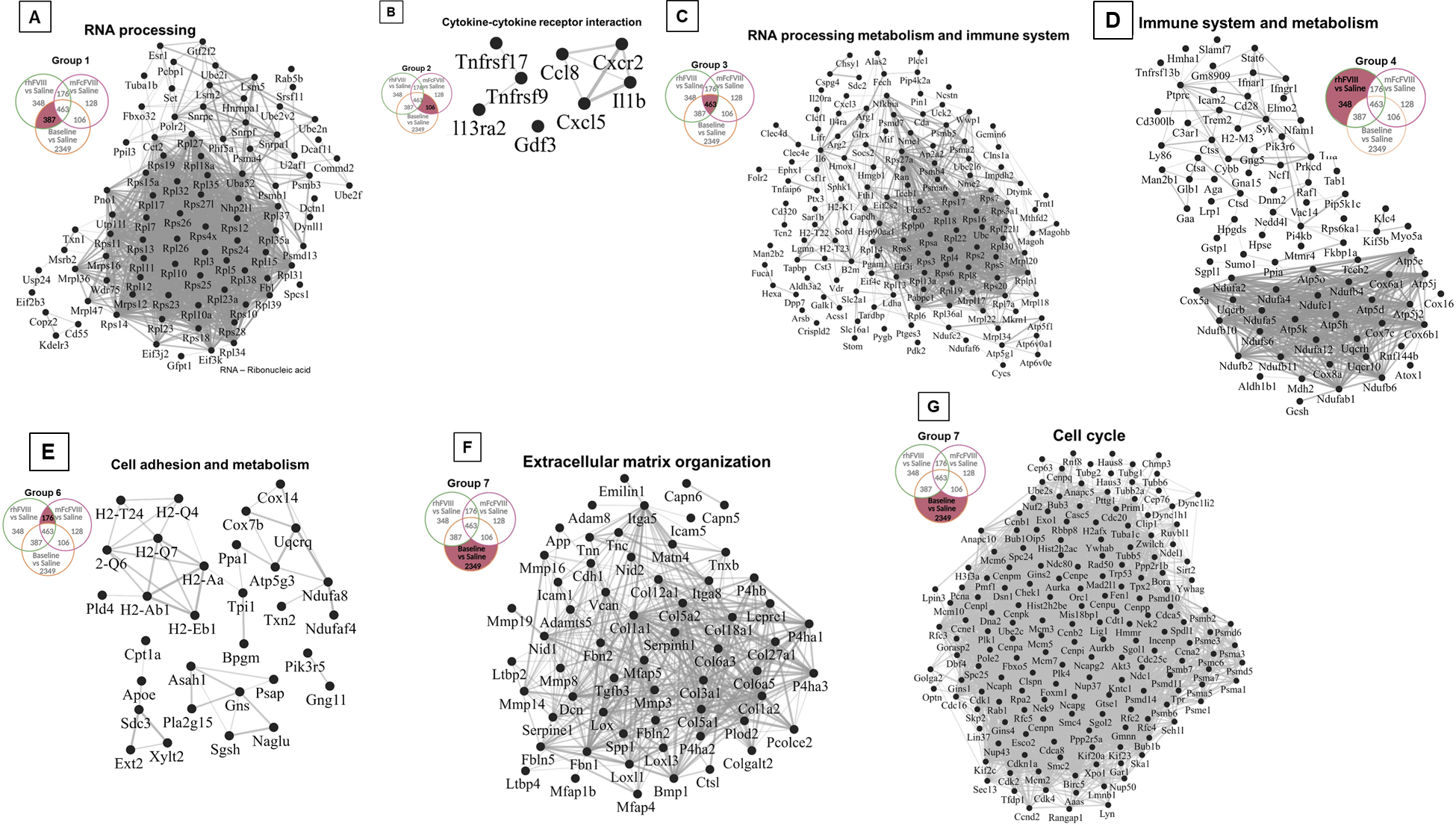

Supplement: S3 Fig — On-target and off-target subsets of DEGs (Figure 2) were subjected to enrichment analysis using gprofiler to identify significant pathways from KEGG and Reactome databases. Representative pathways in each of the subsets are shown. Enriched pathways of group 1 was RNA processing (A). In group 2, the cytokine-cytokine interaction pathway was enhanced (B), in group 3, pathways related to immune system, RNA processing and metabolism were enriched (C). For group 4, metabolism and immune system pathways were enriched (D), and for group 6 cell adhesion and metabolism pathways were enriched (E). Extracellular matrix organization and cell cycle pathway were enriched in group 7 (F and G). DEGs, differentially expressed genes; KEGG, Kyoto Encyclopedia of Genes and Genomes; RNA, ribonucleic acid. (TIF) [file pone.0320322.s003.tif]

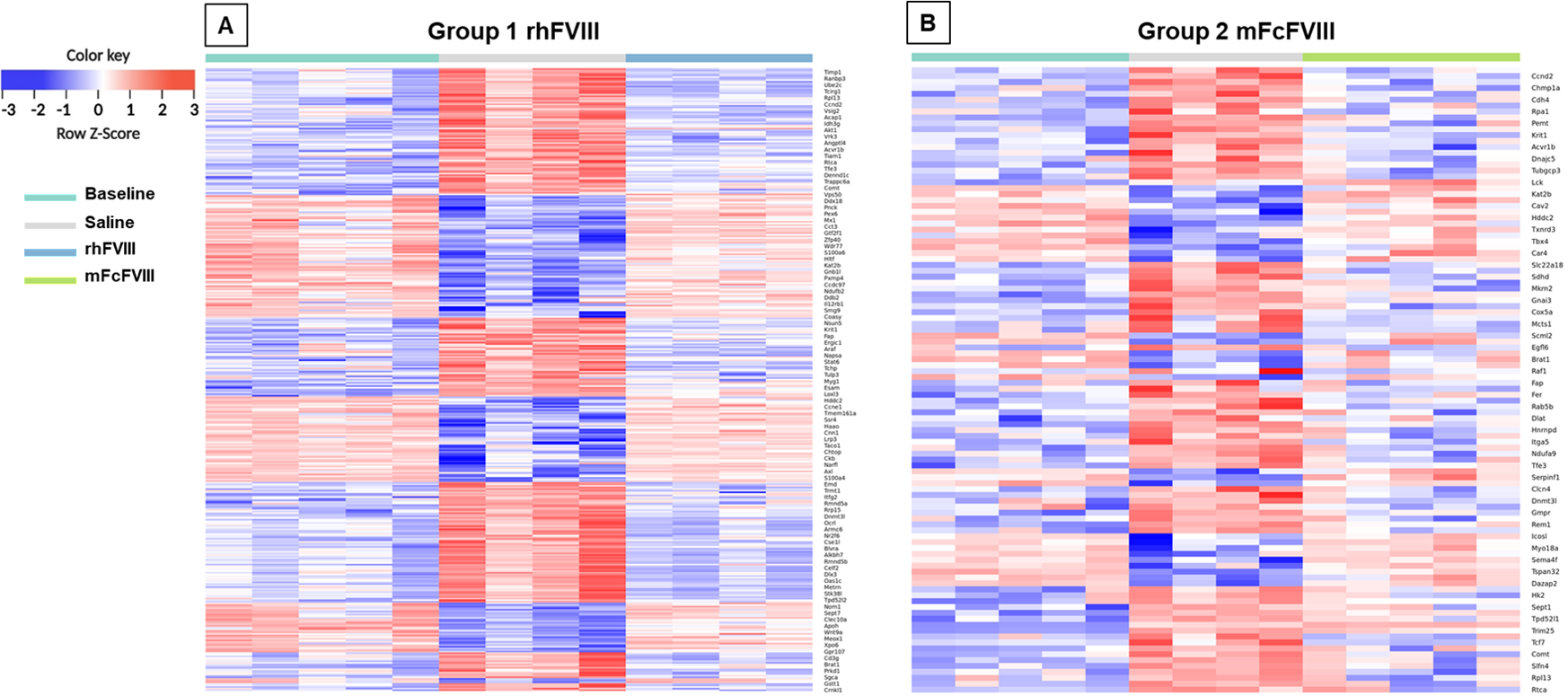

Supplement: S4 Fig — Heatmaps were generated for all the genes in groups 1 and 2 using the R function heatmap.2 with row normalization (Z-score). Each column represents gene expression from an individual mouse in each group: uninjured (baseline), injured-saline, and injured-rhFVIII treated (A), uninjured (baseline), injured-saline, and injured-mFcFVIII treated (B). rhFVIII, recombinant human factor VIII; mFcFVIII, mouse-specific Fc fusion factor VIII. (TIF) [file pone.0320322.s004.tif]

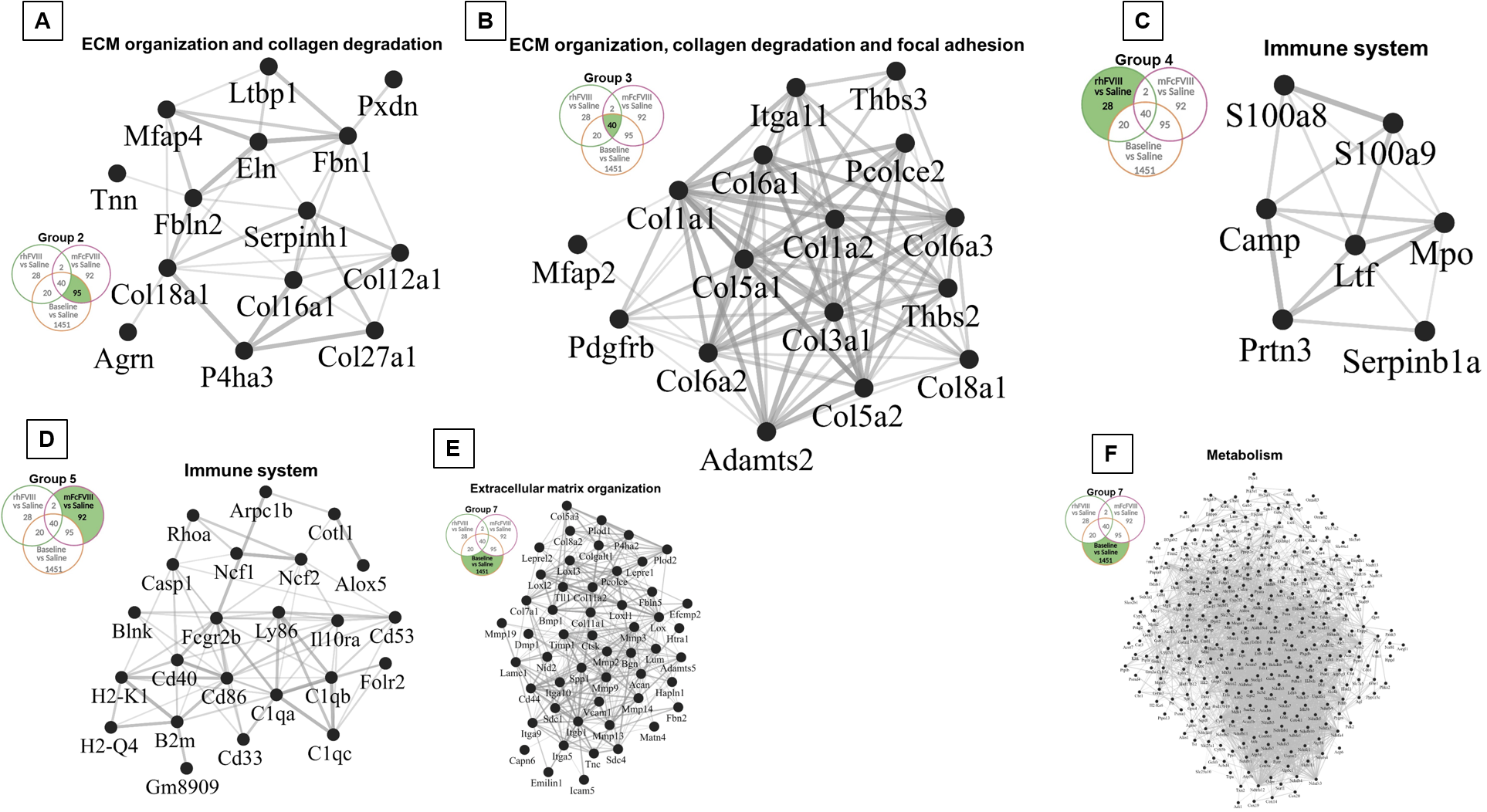

Supplement: S5 Fig — On-target and off-target subsets of DEGs were subjected to enrichment analysis using gprofiler to identify significant pathways from KEGG and Reactome databases. Representative pathways in each of the subsets are shown. Enriched pathways of group 2 were extracellular matrix organization and collagen degradation (A). In group 3 extracellular matrix organization, collagen degradation, and focal adhesion (B) were enriched. Groups 4 and 5 had enriched pathways for the immune system (C and D). Group 7 had enriched pathways predominantly relating to extracellular matrix organization (E) and metabolism (F). DEGs, differentially expressed genes; KEGG, Kyoto Encyclopedia of Genes and Genomes; ECM, extracellular matrix. (TIF) [file pone.0320322.s005.tif]

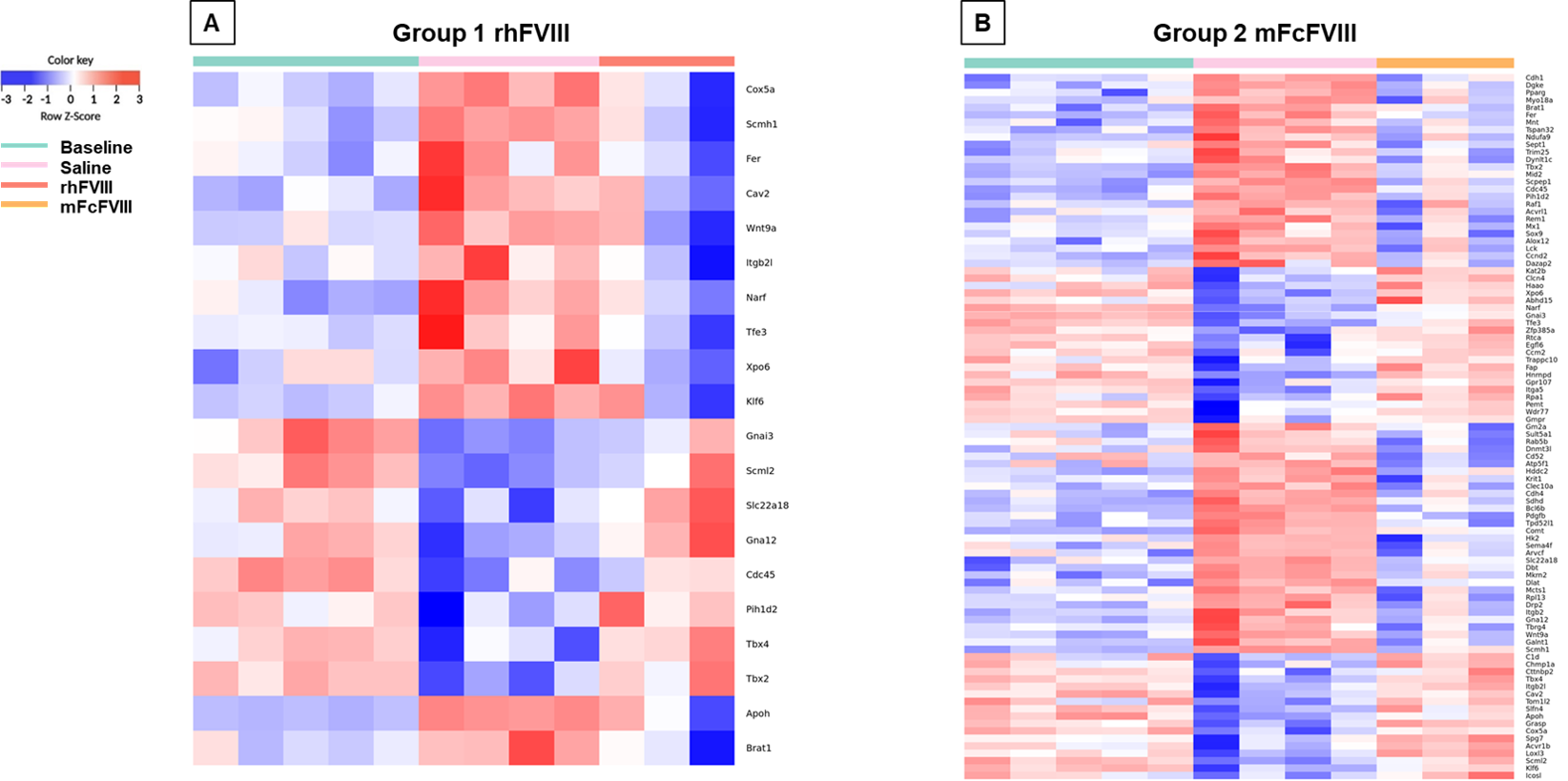

Supplement: S6 Fig — Heatmaps were generated for all the genes in groups 1 and 2 using Rstudio with row normalization (Z-score). Each column represents gene expression from an individual mouse in each group: uninjured (baseline), injured-saline, and injured-rhFVIII treated (A), uninjured (baseline), injured-saline, and injured-mFcFVIII treated (B). rhFVIII, recombinant human factor VIII; mFcFVIII, mouse-specific Fc fusion factor VIII. (TIF) [file pone.0320322.s006.tif]
