# Supplementary material for: Synovial Gene expression after Hemarthrosis differs between FVIII-deficient mice treated with recombinant FVIII or FVIII-Fc Fusion Protein
Source: PLoS One. 2025 May 19;20(5):e0320322. doi: 10.1371/journal.pone.0320322 (PMC12088034; doi:10.1371/journal.pone.0320322)
Supplement: Attachment — Submitted filename: review plus one.docx [file pone.0320322.s011.docx]

This study examines at day 3 and day 14, the effects of murine FVIII-Fc fusion protein (mFcFVIII) in comparison to recombinant human FVIII (rhFVIII) on synovial molecular processes in a context of hemarthrosis. Hemarthrosis were induced in FVII-KO mice by sub-patellar needle puncture and an approach bases on RNAseq and differential gene expression analysis complimented by KEGG and Reactome pathway analysis were applied to synovial tissue. The authors observe a small proportion of genes affected by FVIII treatment and distinct differences between both FVIII concentrates, both related and unrelated to injury/bleed. At day 3, mFcVIII had effects on immune and inflammatory processes whereas rhFVIII seems more modulated mRNA and protein processes. At day 14, molecular profiling revealed expression changes in favour of a transition to synovial tissue repair and remodelling and the authors argue that mFcFVIII compared to rhFVIII facilitated the M1 to M2 macrophage transition. The study, only based on transcriptomic analysis, seems methodologically well conducted, but some issues need to be addressed:

Major remarks:

- The limitations of studies based solely on transcriptomic approaches are not indicated in discussion. These limitations must be added.
- In results section, the authors written at the beginning of the section “injured mice treated with rhFVII vs mFcFVII revealing similar expression profiles without statistical difference” that is in contradiction with the results that follow. Can the authors revise the wording of this sentence to be more explicit?
- The classification of genes/pathways as on-target (related to injury/bleed) and off-target (unrelated to injury/bleed) is not very clear. For example, in results, cytokine-cytokine receptor interaction is on-target and perturbation of immune system is off-target and focal adhesion is on-target and cell adhesion is off-target. This point needs to be clarified.
- In results section, the second paragraph focus on used methodology and should be moved in material and methods section.
- Did the authors apply the cell-type deconvolution from bulk RNASeq using immune cell references at day 3. It would be interesting to present this data.
- Concerning the differential expression levels of macrophage subtype-specific markers M1 (figure 5B), the authors indicated in results section “among the 5 M1 markers, only il6 was mildly increased after saline treatment compared to baseline and decreased with both FVIII-preparations, indicating overall abating inflammation”. The effect observed is very modest and the authors seem to have overinterpreted their results.
- It is the same remark for the discussion about these results (“mFcFVIII facilitate a polarization to M2, not observed to the same extent with rhFVIII)”. Concerning the M2 markers, the difference between rhFVIII and mFcFVIII for lrf4 seems minimal. The last sentence of the results needs to be modified. What are the results for the anti-inflammatory M2 macrophages markers?

Minor remarks:

- The use of abbreviations needs to be reviewed. There should be no abbreviation in the abstract and once an abbreviation is defined in the text it should then be systematically used.
- The title of the Table I is cut.
- In supplementary figures, the figure 1 is illegible for the part on the right.
